# Ethyl (2*E*)-2-cyano-3-(4-meth­oxy­phen­yl)acrylate

**DOI:** 10.1107/S1600536813026871

**Published:** 2013-10-05

**Authors:** P. A. Suchetan, B. S. Palakshamurthy, N. R. Mohan, S. Madan Kumar, N. K. Lokanath, S. Sreenivasa

**Affiliations:** a Department of Studies and Research in Chemistry, U.C.S., Tumkur University, Tumkur, Karnataka 572 103, India; b Department of Studies and Research in Physics, U.C.S., Tumkur University, Tumkur, Karnataka 572 103, India; c Department of Studies and Research in Chemistry, Tumkur University, Tumkur, Karnataka 572 103, India; d Department of Studies in Physics, University of Mysore, Manasagangotri, Mysore, India

## Abstract

In the title compound, C_13_H_13_NO_3_, the conformation across the C=C bond is synperiplanar, the torsion angle of the segment C(ring)—C=C—C(N) being 3.2 (5)°. In the crystal, mol­ecules are linked into inversion dimers, arranged in a zigzag pattern, through two C—H⋯O inter­actions generating *R*_2_^2^(10) and *R*_2_^2^(14) motifs. These dimers are arranged in a zigzag pattern in the crystal structure. The mol­ecules are further linked along the *c* axis through weak C—H⋯π inter­actions, and weak π⋯π inter­actions [centroid–centroid separation = 3.9986 (17) Å] are also observed.

## Related literature

For use of the title compound in the synthesis of prop-2-enoyl­amides, see: Santos *et al.*. (2004 ▶). For use of the title compound in the synthesis of prop-2-enoates, see: Sousa *et al.* (2006 ▶). For hydrogen-bond motifs, see: Bernstein *et al.* (1995 ▶).
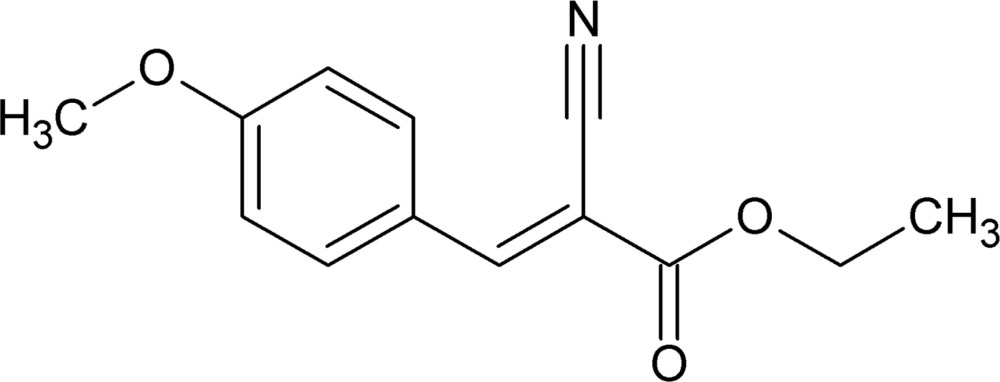


## Experimental

### 

#### Crystal data

C_13_H_13_NO_3_
*M* *_r_* = 231.24
Monoclinic, 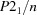

*a* = 8.4889 (12) Å
*b* = 8.3552 (16) Å
*c* = 17.143 (3) Å
β = 91.294 (11)°
*V* = 1215.6 (3) Å^3^
*Z* = 4
Cu *K*α radiation
μ = 0.74 mm^−1^
*T* = 296 K
0.40 × 0.33 × 0.27 mm

#### Data collection

Bruker APEXII CCD diffractometer
4798 measured reflections
1937 independent reflections
1354 reflections with *I* > 2σ(*I*)
*R*_int_ = 0.069

#### Refinement

*R*[*F* ^2^ > 2σ(*F* ^2^)] = 0.067
*wR*(*F* ^2^) = 0.201
*S* = 1.02
1937 reflections
156 parameters
H-atom parameters constrained
Δρ_max_ = 0.18 e Å^−3^
Δρ_min_ = −0.25 e Å^−3^

### 

Data collection: *APEX2* (Bruker, 2009 ▶); cell refinement: *APEX2* and *SAINT-Plus* (Bruker, 2009 ▶); data reduction: *SAINT-Plus* and *XPREP*; program(s) used to solve structure: *SHELXS97* (Sheldrick, 2008 ▶); program(s) used to refine structure: *SHELXL97* (Sheldrick, 2008 ▶); molecular graphics: *Mercury* (Macrae *et al.*, 2008 ▶); software used to prepare material for publication: *SHELXL97*.

## Supplementary Material

Crystal structure: contains datablock(s) I, New_Global_Publ_Block. DOI: 10.1107/S1600536813026871/sj5355sup1.cif

Structure factors: contains datablock(s) I. DOI: 10.1107/S1600536813026871/sj5355Isup2.hkl

Supplementary material file. DOI: 10.1107/S1600536813026871/sj5355Isup3.cml

Additional supplementary materials:  crystallographic information; 3D view; checkCIF report

## Figures and Tables

**Table 1:** Hydrogen-bond geometry (Å, °) *Cg* is the centroid of the C1–C6 benzene ring.

*D*—H⋯*A*	*D*—H	H⋯*A*	*D*⋯*A*	*D*—H⋯*A*
C3—H3⋯O2^i^	0.93	2.54	3.414 (3)	157
C8—H8⋯O2^i^	0.93	2.59	3.475 (3)	159
C12—H12*A*⋯*Cg* ^ii^	0.97	2.90	3.803 (3)	156

Symmetry codes: (i) 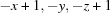; (ii) 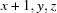.

**Fig. 1.**
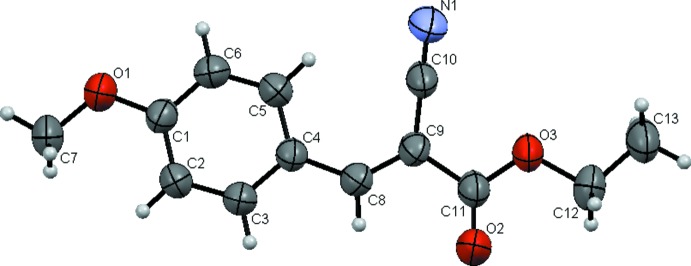
Molecular structure of the title compound, showing the atom-labeling scheme. Displacement ellipsoids are drawn at the 50% probability level.

**Fig. 2.**
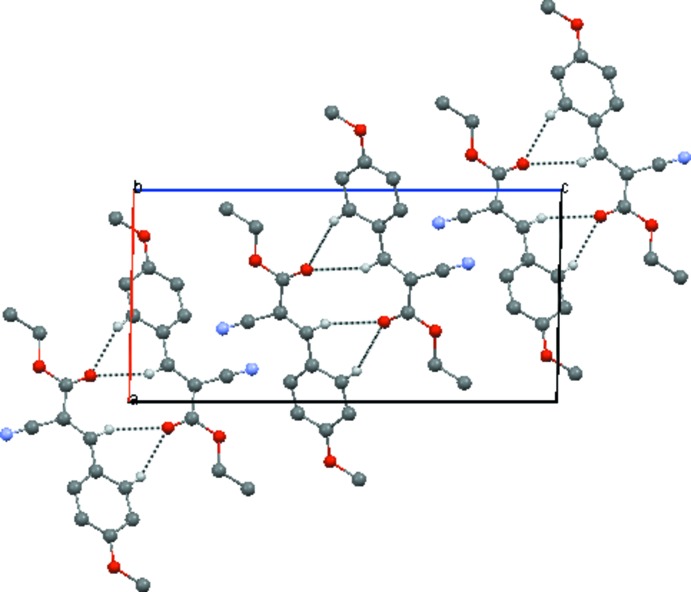
Molecular packing in the title compound displaying R22(10) and R22(14)rings chains. H atoms not involved in H-bonding are omitted for clarity.

**Fig. 3.**
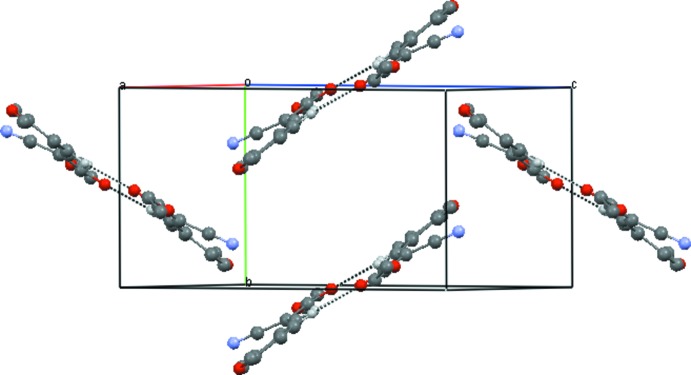
The zigzag pattern of inversion dimers viewed along a.

**Fig. 4.**
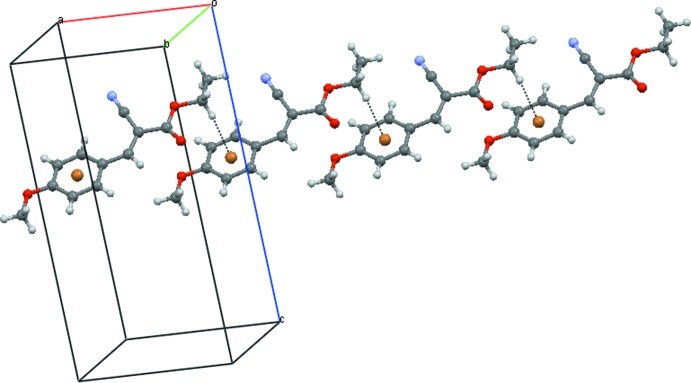
Linking of molecules along the c axis through C—H···Cg interactions.

**Fig. 5.**
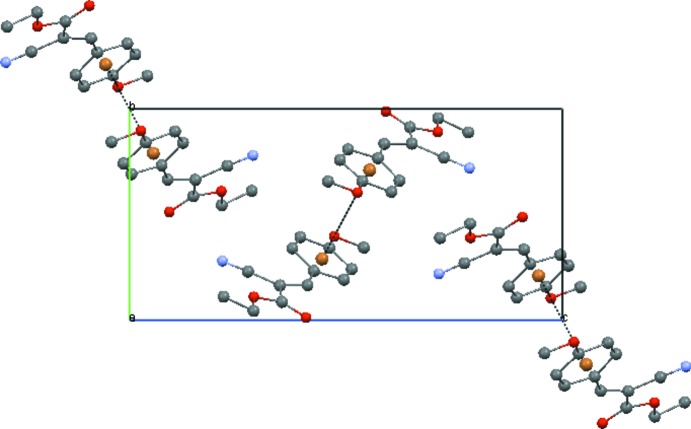
π-π stacking interactions observed in the crystal structure. H atoms are omitted for clarity.

## supplementary crystallographic information

### 1. Comment

Ethyl (2E)-2-cyano-3-(4-methoxyphenyl)acrylate is an important starting material for the synthesis of biologically and pharmacologically important 2-propenoylamides (Santos *et al.*., 2004) and 2-propenoates (Sousa *et al*., 2006). Keeping this in mind, the title compound was synthesized.

In the title compound, C_13_H_13_NO_3_, the conformation across the C=C bond is *syn*-periplanar (Fig.1), the C4—C8—C9—C10 torsion angle being 3.2 (5)°. The molecules are linked into inversion dimers (Fig.2) through C3—H3···O2 and C8—H8···O2 interactions, generating *R*_2_^2^(10) and *R*_2_^2^(14) ring motifs (Bernstein *et al.*, 1995). These dimers are arranged in a zigzag pattern in the crystal structure (Fig.3). The molecules are further linked along the *c* axis through weak C12—H12A···*Cg* interactions (Fig.4), and weak *Cg*···*Cg* interactions (centroid···centroid separation = 3.9986 (17) Å) are also observed (Fig.5).

### 2. Experimental

A mixture of tetra-*n*-butylammonium bromide (TBAB) (3.47 g, 10 mmol), palladium acetate (0.060 g, 0.27 mmol), triphenylphosphine (2.97 g, 11 mmol), 1-bromo-4-methoxybenzene (1 g, 5.4 mmol) and potassium carbonate (1.12 g, 8.1 mmol) were dissolved in 10 ml of DMF in a three-necked round bottom flask and the reaction mixture was heated to 120 °C under nitrogen atmosphere with constant stirring for 10–15 minutes until a yellowish brown solution was obtained. Ethyl-2-cyanoacrylate (0.81 g, 6.4 mmol) was added to the solution and the reaction mixture was heated to 120 °C for 10 h, cooled and filtered under vacuum to obtain the crude compound. This was further purified by column chromatography using petroleum ether: ethyl acetate (7:3) as eluent (Rf value = 0.69), to yield pale green colored crystals.

### 3. Refinement

The H atoms were positioned with idealized geometry using a riding model with C—H = 0.93–0.97 Å. All H atoms were refined with isotropic displacement parameters (set to 1.2–1.5 times *U*_eq_ of the parent atom).

### Crystal data

**Table:**

C_13_H_13_NO_3_	Prism
*M**_r_* = 231.24	*D*_x_ = 1.264 Mg m^−^^3^
Monoclinic, *P*2_1_/*n*	Melting point: 401 K
Hall symbol: -P 2yn	Cu *K*α radiation, λ = 1.54178 Å
*a* = 8.4889 (12) Å	Cell parameters from 776 reflections
*b* = 8.3552 (16) Å	θ = 5.2–64.3°
*c* = 17.143 (3) Å	µ = 0.74 mm^−^^1^
β = 91.294 (11)°	*T* = 296 K
*V* = 1215.6 (3) Å^3^	Prism, green
*Z* = 4	0.40 × 0.33 × 0.27 mm
*F*(000) = 488	

### Data collection

**Table:**

Bruker APEXII CCD diffractometer	1354 reflections with *I* > 2σ(*I*)
Radiation source: fine-focus sealed tube	*R*_int_ = 0.069
Graphite monochromator	θ_max_ = 64.3°, θ_min_ = 5.2°
Detector resolution: 1 pixels mm^-1^	*h* = −9→3
φ and ω scans	*k* = −9→9
4798 measured reflections	*l* = −19→19
1937 independent reflections	

### Refinement

**Table:**

Refinement on *F*^2^	Primary atom site location: structure-invariant direct methods
Least-squares matrix: full	Secondary atom site location: difference Fourier map
*R*[*F*^2^ > 2σ(*F*^2^)] = 0.067	Hydrogen site location: inferred from neighbouring sites
*wR*(*F*^2^) = 0.201	H-atom parameters constrained
*S* = 1.02	*w* = 1/[σ^2^(*F*_o_^2^) + (0.124*P*)^2^] where *P* = (*F*_o_^2^ + 2*F*_c_^2^)/3
1937 reflections	(Δ/σ)_max_ = 0.012
156 parameters	Δρ_max_ = 0.18 e Å^−^^3^
0 restraints	Δρ_min_ = −0.25 e Å^−^^3^

### Special details

**Table:**

Geometry. All s.u.'s (except the s.u. in the dihedral angle between two l.s. planes) are estimated using the full covariance matrix. The cell s.u.'s are taken into account individually in the estimation of s.u.'s in distances, angles and torsion angles; correlations between s.u.'s in cell parameters are only used when they are defined by crystal symmetry. An approximate (isotropic) treatment of cell s.u.'s is used for estimating s.u.'s involving l.s. planes.
Refinement. Refinement of *F*^2^ against ALL reflections. The weighted *R*-factor *wR* and goodness of fit *S* are based on *F*^2^, conventional *R*-factors *R* are based on *F*, with *F* set to zero for negative *F*^2^. The threshold expression of *F*^2^ > 2σ(*F*^2^) is used only for calculating *R*-factors(gt) *etc*. and is not relevant to the choice of reflections for refinement. *R*-factors based on *F*^2^ are statistically about twice as large as those based on *F*, and *R*- factors based on ALL data will be even larger.

### Fractional atomic coordinates and isotropic or equivalent isotropic displacement parameters (Å^2^)

**Table:**

	*x*	*y*	*z*	*U*_iso_*/*U*_eq_	
C1	1.1331 (3)	0.3418 (3)	0.46205 (14)	0.0559 (7)	
C2	1.0569 (3)	0.2415 (4)	0.51365 (14)	0.0599 (7)	
H2	1.1067	0.2089	0.5599	0.072*	
C3	0.9066 (3)	0.1912 (4)	0.49517 (14)	0.0597 (7)	
H3	0.8547	0.1266	0.5305	0.072*	
C4	0.8284 (3)	0.2330 (3)	0.42574 (13)	0.0510 (7)	
C5	0.9065 (3)	0.3398 (4)	0.37625 (14)	0.0593 (7)	
H5	0.8566	0.3743	0.3304	0.071*	
C6	1.0549 (3)	0.3931 (4)	0.39481 (14)	0.0621 (7)	
H6	1.1040	0.4650	0.3618	0.074*	
C7	1.3670 (3)	0.3435 (5)	0.54148 (17)	0.0817 (10)	
H7A	1.3118	0.3761	0.5871	0.122*	
H7B	1.4705	0.3899	0.5425	0.122*	
H7C	1.3756	0.2289	0.5407	0.122*	
C8	0.6735 (3)	0.1658 (3)	0.41146 (14)	0.0550 (7)	
H8	0.6320	0.1138	0.4543	0.066*	
C9	0.5784 (3)	0.1645 (3)	0.34770 (14)	0.0540 (7)	
C10	0.6207 (3)	0.2301 (4)	0.27376 (14)	0.0639 (8)	
C11	0.4219 (3)	0.0864 (4)	0.35228 (14)	0.0577 (7)	
C12	0.1752 (3)	0.0439 (4)	0.28684 (17)	0.0698 (8)	
H12A	0.1221	0.0730	0.3343	0.084*	
H12B	0.1790	−0.0720	0.2835	0.084*	
C13	0.0891 (3)	0.1113 (5)	0.21740 (18)	0.0856 (11)	
H13A	0.0878	0.2259	0.2209	0.128*	
H13B	−0.0171	0.0717	0.2159	0.128*	
H13C	0.1413	0.0795	0.1708	0.128*	
N1	0.6549 (3)	0.2803 (5)	0.21466 (14)	0.0962 (11)	
O1	1.2826 (2)	0.3962 (3)	0.47346 (11)	0.0737 (7)	
O2	0.3775 (2)	0.0120 (3)	0.40764 (12)	0.0812 (7)	
O3	0.33402 (18)	0.1098 (3)	0.28756 (9)	0.0649 (6)	

### Atomic displacement parameters (Å^2^)

**Table:**

	*U*^11^	*U*^22^	*U*^33^	*U*^12^	*U*^13^	*U*^23^
C1	0.0466 (14)	0.0676 (18)	0.0535 (13)	−0.0088 (12)	0.0015 (10)	−0.0033 (12)
C2	0.0525 (14)	0.078 (2)	0.0488 (13)	−0.0068 (13)	−0.0061 (10)	0.0058 (12)
C3	0.0525 (14)	0.0777 (19)	0.0489 (13)	−0.0081 (13)	−0.0002 (10)	0.0077 (12)
C4	0.0431 (13)	0.0640 (16)	0.0458 (12)	−0.0001 (11)	0.0003 (9)	−0.0013 (11)
C5	0.0536 (15)	0.0757 (19)	0.0486 (13)	−0.0014 (13)	−0.0028 (10)	0.0082 (12)
C6	0.0575 (15)	0.077 (2)	0.0520 (13)	−0.0105 (14)	0.0036 (11)	0.0077 (13)
C7	0.0549 (16)	0.118 (3)	0.0713 (18)	−0.0178 (17)	−0.0162 (13)	0.0095 (17)
C8	0.0470 (13)	0.0680 (18)	0.0501 (13)	0.0007 (12)	0.0017 (10)	0.0037 (11)
C9	0.0402 (13)	0.0707 (17)	0.0512 (13)	0.0026 (11)	0.0027 (10)	−0.0018 (11)
C10	0.0443 (13)	0.099 (2)	0.0477 (14)	0.0013 (14)	−0.0023 (10)	−0.0015 (14)
C11	0.0454 (13)	0.0742 (19)	0.0534 (13)	0.0030 (12)	−0.0029 (11)	−0.0020 (13)
C12	0.0458 (15)	0.079 (2)	0.0841 (18)	−0.0014 (13)	−0.0088 (13)	−0.0056 (15)
C13	0.0530 (16)	0.128 (3)	0.0748 (19)	0.0064 (17)	−0.0112 (13)	−0.0004 (19)
N1	0.0689 (16)	0.167 (3)	0.0526 (14)	−0.0126 (17)	0.0023 (11)	0.0125 (16)
O1	0.0546 (11)	0.0997 (17)	0.0665 (11)	−0.0192 (10)	−0.0074 (8)	0.0085 (11)
O2	0.0605 (12)	0.1123 (19)	0.0705 (12)	−0.0192 (11)	−0.0087 (9)	0.0250 (12)
O3	0.0449 (10)	0.0925 (16)	0.0570 (10)	−0.0033 (9)	−0.0067 (8)	0.0006 (9)

### Geometric parameters (Å, º)

**Table:**

C1—O1	1.358 (3)	C7—H7C	0.9600
C1—C6	1.385 (4)	C8—C9	1.344 (3)
C1—C2	1.389 (4)	C8—H8	0.9300
C2—C3	1.374 (3)	C9—C10	1.434 (4)
C2—H2	0.9300	C9—C11	1.483 (4)
C3—C4	1.394 (3)	C10—N1	1.140 (3)
C3—H3	0.9300	C11—O2	1.203 (3)
C4—C5	1.407 (4)	C11—O3	1.337 (3)
C4—C8	1.446 (3)	C12—O3	1.456 (3)
C5—C6	1.367 (4)	C12—C13	1.493 (4)
C5—H5	0.9300	C12—H12A	0.9700
C6—H6	0.9300	C12—H12B	0.9700
C7—O1	1.425 (3)	C13—H13A	0.9600
C7—H7A	0.9600	C13—H13B	0.9600
C7—H7B	0.9600	C13—H13C	0.9600
			
O1—C1—C6	116.4 (2)	C9—C8—C4	131.9 (2)
O1—C1—C2	123.9 (2)	C9—C8—H8	114.0
C6—C1—C2	119.7 (2)	C4—C8—H8	114.0
C3—C2—C1	118.8 (2)	C8—C9—C10	123.9 (2)
C3—C2—H2	120.6	C8—C9—C11	118.9 (2)
C1—C2—H2	120.6	C10—C9—C11	117.2 (2)
C2—C3—C4	122.8 (2)	N1—C10—C9	179.1 (4)
C2—C3—H3	118.6	O2—C11—O3	123.4 (2)
C4—C3—H3	118.6	O2—C11—C9	124.5 (2)
C3—C4—C5	116.9 (2)	O3—C11—C9	112.1 (2)
C3—C4—C8	117.4 (2)	O3—C12—C13	107.5 (3)
C5—C4—C8	125.7 (2)	O3—C12—H12A	110.2
C6—C5—C4	120.7 (2)	C13—C12—H12A	110.2
C6—C5—H5	119.6	O3—C12—H12B	110.2
C4—C5—H5	119.6	C13—C12—H12B	110.2
C5—C6—C1	121.0 (2)	H12A—C12—H12B	108.5
C5—C6—H6	119.5	C12—C13—H13A	109.5
C1—C6—H6	119.5	C12—C13—H13B	109.5
O1—C7—H7A	109.5	H13A—C13—H13B	109.5
O1—C7—H7B	109.5	C12—C13—H13C	109.5
H7A—C7—H7B	109.5	H13A—C13—H13C	109.5
O1—C7—H7C	109.5	H13B—C13—H13C	109.5
H7A—C7—H7C	109.5	C1—O1—C7	117.7 (2)
H7B—C7—H7C	109.5	C11—O3—C12	116.8 (2)
			
O1—C1—C2—C3	−178.6 (3)	C4—C8—C9—C10	3.2 (5)
C6—C1—C2—C3	2.1 (4)	C4—C8—C9—C11	−178.8 (3)
C1—C2—C3—C4	1.8 (4)	C8—C9—C11—O2	−6.2 (5)
C2—C3—C4—C5	−4.2 (4)	C10—C9—C11—O2	171.9 (3)
C2—C3—C4—C8	176.9 (2)	C8—C9—C11—O3	173.1 (2)
C3—C4—C5—C6	2.8 (4)	C10—C9—C11—O3	−8.8 (4)
C8—C4—C5—C6	−178.5 (3)	C6—C1—O1—C7	−179.0 (3)
C4—C5—C6—C1	1.0 (4)	C2—C1—O1—C7	1.7 (4)
O1—C1—C6—C5	177.2 (3)	O2—C11—O3—C12	1.7 (4)
C2—C1—C6—C5	−3.5 (4)	C9—C11—O3—C12	−177.6 (2)
C3—C4—C8—C9	−169.8 (3)	C13—C12—O3—C11	168.8 (3)
C5—C4—C8—C9	11.4 (5)		

### Hydrogen-bond geometry (Å, º)

Cg is the centroid of the C1···C6 benzene ring.

**Table:**

*D*—H···*A*	*D*—H	H···*A*	*D*···*A*	*D*—H···*A*
C3—H3···O2^i^	0.93	2.54	3.414 (3)	157
C8—H8···O2^i^	0.93	2.59	3.475 (3)	159
C12—H12*A*···*Cg*^ii^	0.97	2.90	3.803 (3)	156

Symmetry codes: (i) −*x*+1, −*y*, −*z*+1; (ii) *x*+1, *y*, *z*.
